# Swedish intrauterine growth reference ranges for estimated fetal weight

**DOI:** 10.1038/s41598-021-92032-2

**Published:** 2021-06-14

**Authors:** Linda Lindström, Mårten Ageheim, Ove Axelsson, Laith Hussain-Alkhateeb, Alkistis Skalkidou, Anna-Karin Wikström, Eva Bergman

**Affiliations:** 1 Department of Women’s and Children’s Health, Uppsala University, Uppsala, Sweden; 2 Centre for Clinical Research Sörmland, Uppsala University, Eskilstuna, Sweden; 3 Global Health, School of Public Health and Community Medicine, Institute of Medicine, Sahlgrenska Academy, University of Gothenburg, Gothenburg, Sweden

**Keywords:** Public health, Intrauterine growth

## Abstract

Fetal growth restriction is a strong risk factor for perinatal morbidity and mortality. Reliable standards are indispensable, both to assess fetal growth and to evaluate birthweight and early postnatal growth in infants born preterm. The aim of this study was to create updated Swedish reference ranges for estimated fetal weight (EFW) from gestational week 12–42. This prospective longitudinal multicentre study included 583 women without known conditions causing aberrant fetal growth. Each woman was assigned a randomly selected protocol of five ultrasound scans from gestational week 12 + 3 to 41 + 6. Hadlock’s 3rd formula was used to estimate fetal weight. A two-level hierarchical regression model was employed to calculate the expected median and variance, expressed in standard deviations and percentiles, for EFW. EFW was higher for males than females. The reference ranges were compared with the presently used Swedish, and international reference ranges. Our reference ranges had higher EFW than the presently used Swedish reference ranges from gestational week 33, and higher median, 2.5th and 97.5th percentiles from gestational week 24 compared with INTERGROWTH-21st. The new reference ranges can be used both for assessment of intrauterine fetal weight and growth, and early postnatal growth in children born preterm.

## Introduction

An important part of antenatal care is assessment of fetal growth, as growth and birthweight are important factors that influence perinatal morbidity and mortality^1–3^. There are different approaches in screening for fetuses with high risk of morbidity and mortality due to aberrant growth. Serial measurements of the symphysis-fundus distance can be used as a simple and inexpensive screening tool, followed by ultrasonic estimation of fetal size if deviating^4–6^. Selective or universal ultrasonography are other screening tools for aberrant fetal growth. Irrespective of indication, reliable standards are vital for the evaluation of fetal size and timely recognition of aberrant growth.

A high-quality standard for fetal size and growth describes how fetuses grow under optimal conditions. Reference ranges of intrauterine growth should be based on studies with a robust design, a reasonably large cohort and use adequate statistical modelling methods for calculation of percentiles, to aid clinical judgement^7,8^. Children born preterm are more often growth restricted compared with children born term^9^. Thereby, the birthweight of children born preterm is substantially lower than the weight in children of the same gestational age (GA) who remain in utero until term^10–12^. Thus, relying on information of birthweight in infants born preterm to extrapolate and develop fetal weight reference ranges should be avoided; rather longitudinal intrauterine assessment of fetal weight should be used^10,12,13^. Moreover, the inevitable neonatal intensive care exposes the preterm born infant to a stressful extrauterine environment that affect early postnatal growth, and consequently intrauterine growth reference ranges are often recommended for monitoring early postnatal growth in preterm born infants^13^.

Fetal weight can be estimated using a variety of algorithms, which combine ultrasonographic assessment of biometric measurements. The most widely used algorithms include combinations of biparietal diameter (BPD), head circumference (HC), mean abdominal diameter (MAD), abdominal circumference (AC) and femur length (FL)^14^. Several studies have evaluated these algorithms, as there is no universally accepted formula for fetal weight estimation^15–18^. Even though the results diverge to some extent, most studies have found Hadlock’s formula, including HC, AC and FL with or without addition of BPD, superior or non-inferior to other formulae in estimating fetal weight or predicting adverse perinatal outcome^15–19^.

During the last years, the World Health Organization Multicentre Growth Reference Study and the INTERGROWTH-21st have presented fetal growth standards intended for universal use in all populations^20,21^. Due to variations in fetal size over the globe, it has been questioned if these international standards are better than national references in detecting growth restricted fetuses with increased risk of adverse outcome^22–27^.

The currently used Swedish growth reference ranges for estimated fetal weight (EFW) were published in 1996 by Maršál et al., and present EFW from gestational week 25 to 43. The growth reference ranges are based on 86 Scandinavian pregnant women, of whom 24% were smokers^10^. Today, fetal interventions can be made during the second trimester of pregnancy, and survival is possible for children born as early as 22 gestational weeks. To meet up with these obstetrical challenges, there is a need for updated national reference ranges, which reflect expected fetal growth throughout the second trimester in a representative and healthy population.

The aim of this study was to create new national reference ranges for EFW from gestational week 12 to 42, applying modern statistical methods to a longitudinal study of ultrasonically derived intrauterine biometric measurements in a large Swedish cohort of healthy pregnant women with expected normal fetal growth. Further, we wanted to compare the new reference ranges with other relevant growth reference ranges of EFW.

## Methods

### Study design and population

In this prospective longitudinal multicenter study, 684 women were recruited in early pregnancy between September 2015 and September 2018 in five sites in central Sweden; Uppsala, Falun, Katrineholm, Västerås and Örebro. In Sweden, antenatal care is standardized and free of charge. All women are offered a routine second trimester ultrasound scan. The purpose of this scan is to evaluate GA (unless the pregnancy was dated during the first trimester), number of fetuses, viability, placental location and search for structural anomalies^28^. Women with low risk pregnancies are not routinely scanned for aberrant fetal growth with ultrasound during the third trimester of pregnancy. At first antenatal visit, all women who received antenatal care at 18 primary care units in urban as well as rural areas representing different socioeconomic and ethnic backgrounds were invited to participate in the study. Non-smoking women were eligible if they had regular menstrual periods (28 ± 4 days), a spontaneously conceived pregnancy, and no previous pregnancy complications, such as preterm birth, gestational hypertension, pre-eclampsia or eclampsia, gestational diabetes or stillbirth. Women with chronic hypertension, systemic lupus erythematosus, kidney disease, diabetes mellitus, previous gastric bypass surgery or inflammatory bowel disease were not eligible, as these conditions are known to affect fetal growth.

Eligible women were invited to the first study visit between gestational week 12 + 3 and 13 + 6 according to the last menstrual period. Eligibility was evaluated and GA assessed with ultrasound. Only women with singleton pregnancies with BPD ≥ 21 mm were recruited, and GA according to BPD should not deviate more than seven days from GA according to last menstrual period. Written informed consent was obtained from all the study subjects before the dating scan was performed.

To get an even distribution of the ultrasound scans throughout pregnancy, each study subject was randomized to one of nine study protocols, with different timing of the ultrasound scans. Each protocol included five scans allocated between gestational week 14 and 41. The study protocols were kept in closed envelopes and were randomly assigned to each woman at inclusion. In Sweden, all pregnant women are offered the routine second trimester scan at gestational week 17–20. If the woman was not randomized to a scan in week 17–20 (five protocols), the biometric measurements were recorded in the study database only if the routine second trimester scan was performed by a study sonographer. In order to compensate for the expected decline in the number of women with ongoing pregnancies, more women were assigned to the scans in gestational week 37–41.

Women were excluded from the study if the pregnancy was complicated by gestational hypertension, pre-eclampsia or eclampsia, gestational diabetes, single umbilical artery, placental complication such as placenta previa and placental abruption, fetal malformations or chromosomal aberrations, stillbirth, fetal growth restriction or preterm birth (before 37 + 0 gestational weeks; 259 gestational days). Fetal growth restriction was defined as EFW < −2 standard deviations (SD) according to the reference ranges by Maršál et al. with abnormal umbilical Doppler and/or oligohydramniosis (single deepest pocket < 2 cm)^10^.

Out of the 684 recruited women, 650 were eligible for the study. During pregnancy, 14 women (2.2%) were excluded due to gestational hypertension or pre-eclampsia, and 11 (1.7%) due to development of gestational diabetes. Six women (0.9%) were excluded as they developed fetoplacental complications, such as placenta previa, placental abruption, single umbilical artery and preterm fetal growth restriction. One woman had a late miscarriage, one child was stillborn and 26 children (4.0%) were born preterm. Lastly, 8 women (1.2%) were excluded due to fetal malformations or chromosomal aberrations. Women who were excluded from the study due to pregnancy complications more often had an increased BMI; 9.9% for BMI 25.0–29.9 kg/m^2^, 23.8% for BMI ≥ 30 kg/m^2^, versus 7.7% for BMI < 25 kg/m^2^, or were born outside the Nordic countries; 15% versus 10%. The final cohort consisted of 583 women; 275 from Uppsala, 66 from Falun, 98 from Katrineholm, 50 from Västerås and 94 from Örebro.

### Procedures

All ultrasound scans were performed by seven experienced sonographers. The ultrasound machines used were GE Voluson E10, GE Voluson E8 and GE Voluson E6 with abdominal transducers 2–6 MHz RM6C, 2–8 MHz C4-8-D, RAB 4–8-D and 2–9 MHz C2-9-D.

Before first inclusion, all sonographers were given detailed oral and written instructions regarding how the biometric measurements should be performed. A repeatability and reproducibility study was performed, which is described in detail in a previous publication^29^. BPD was used to calculate the GA, using the modified Selbing and Kjessler formula, 58.65 + 1.07 * BPD + 0.0138 * BPD^2^, as recommended by the Swedish Society of Obstetrics and Gynecology^30–32^. At each ultrasound scan, the sonographer measured five biometric measurements trifold; BPD, HC, MAD, AC and FL, and all three measurements were registered in the data base. All data was manually registered in a web-based study database by the sonographer.

To estimate fetal weight, we used Hadlock et al.’s formula 3, which includes the biometric measurements BPD, HC, AC and FL^19^. BPD and HC were measured in axial section, at the level of the thalami, with the midline echo in a central position broken anteriorly by cavum septum pellucidum. Orbitae and cerebellum should be non-visible. The callipers for BPD were placed on the outer margin of the proximal parietal bone, and the inner margin of the distal parietal bone. HC was measured by placing the callipers on the outer borders of the frontal and occipital edges of the bone, and the ellipse facility was used to follow the contours of the skull. AC was measured in cross-section, with a circular view of the abdomen, the stomach visible, the umbilical vein in the anterior third of the abdomen and the aorta and inferior vena cava anterior to the spine. Further, the majority of a rib should be seen, but not the heart or kidneys. AC was measured using the ellipse facility to follow the outer contours of the skin. FL was measured in a longitudinal section of the femur in 45°–90° angle of insonation, with the callipers placed on the outer borders of the femoral diaphysis. All measurements followed the national recommendations for biometric assessment and the practice guidelines from The International Society of Ultrasound in Obstetrics and Gynecology^30,33^.

### Data management

Each biometric measurement was estimated three times at each ultrasound scan and registered in the study database, in total 38,601 repeated measurements were included. Data was cleaned in several steps, described in detail in the publication of the individual biometric measurements^29^. In summary, data cleaning included identification of outliers, correction of erroneous data entry and deletion of unreasonable measurements.

### Statistical analysis

Descriptive statistics were used for maternal characteristics at baseline, and for delivery and neonatal characteristics. Mean birthweight was compared in subgroups of the cohort based on country of birth and study site using an independent samples t-test for country of birth and a one-way ANOVA for study site, and *p* value < 0.05 was considered as statistically significant. The discrepancy between mean EFW and actual birthweight was compared using a paired samples t-test in women who delivered within two days after the last ultrasound scan.

Data from the biometric measurements was used to construct reference ranges for BPD, HC, AC and FL^29^. The estimated fetal biometric measurements at each gestational day were used for estimating fetal weight using Hadlock’s 3rd formula^19^. In order to account for the violated normality assumptions of the data, the fetal growth measurements were log-transformed and modelled using a fixed and random effect multilevel regression methods^34^. Fractional polynomial regression was first applied on the log transformed fetal measurement to define best fitting combination of fractional polynomials for the GA. To further illustrate the powers used, the fetal weight had a combination of 0.5 and 3 as the best fitting fractional polynomial powers. These parameters were then entered a fixed effect multilevel regression model to address the repeated measurements nature of the data reported for each fetus^35,36^. A two-level hierarchical model was sought for the repeated measurements of each fetus at each visit with a random effect for the identified fractional polynomial of GA and the intercept. The method of Johnsen et al. was employed to estimate the expected fetal measurements at each GA in weeks and compute their standard deviations (SD) and percentiles^36^.

In a sensitivity analysis, we excluded women with underweight (BMI < 18.5 kg/m^2^) or obesity (BMI ≥ 30.0 kg/m^2^) according to the World Health Organization (WHO) at first antenatal visit. We applied the same adjusted statistical models to the subset of the study cohort to estimate the expected fetal weight at each GA in weeks, and to compute the SD and percentiles. The reference ranges before and after exclusion of women with abnormal BMI were compared using an independent samples t-test, for all subjects as well as stratified according to offspring sex.

The median, − 2 SD and + 2 SD of the new reference ranges were compared with the corresponding values from the current Swedish reference, published by Maršál et al^10^. Further, the median, 2.5th and 97.5th percentiles were compared with the corresponding values from the Norwegian reference ranges more recently published by Johnsen et al. and the international standards published by the INTERGROWTH-21st project and WHO, respectively^20,21,36^.

### Research involving human participants

The study was approved by the Regional Ethical Review Board in Uppsala (Nos. 2014/209 and 2014/209/2). All procedures involving human subjects were carried out in accordance with the ethical standards of the 1964 Helsinki declaration. All women participated voluntarily and gave their written informed consent. Any pregnancy complication recognized during the study was reported to the routine obstetric care at each study site and managed according to clinical practice.

## Results

**Figure 1:**
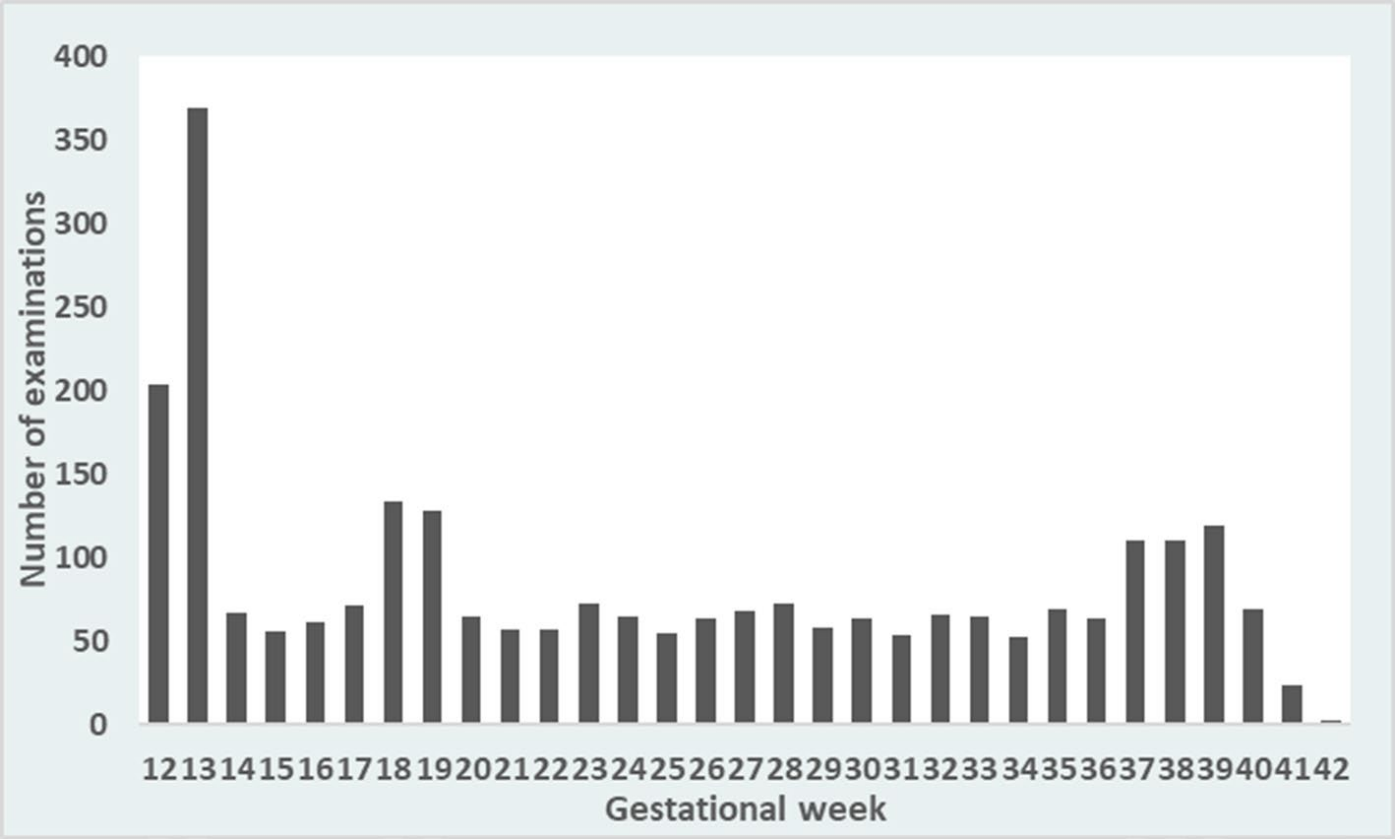
Distribution of ultrasound examinations by gestational age.

In total 2590 ultrasound scans were performed, of which 2551 were complete with data on BPD, HC, AC and FL and hence used for estimating fetal weight. The majority of the women, 526 of 583 (90%), were scanned at least four times. In 187 women (32%), the randomization protocol was completed with five scans evenly distributed over the second and third trimester of pregnancy. In 57 women (10%), only two or three scans were performed. In 145 women (25%), a scan was performed and registered outside the randomization protocol. These scans corresponded to the routine second trimester scan in 139 women, and the remaining 6 scans were performed in gestational week 22, 26, 30, 32, and 35, respectively. The scans following the dating scan were fairly equally distributed over the GAs, see Fig. 1. There was a peak at week 18–19, corresponding to the routine ultrasound scan, and week 37–39. The mean dating discrepancy, i.e. the difference between estimated date of delivery according to BPD at first study visit and estimated date of delivery according to last menstrual period, was − 0.1 days (SD 2.8 days) and the median discrepancy was 0 days. The dating discrepancy was slightly, but not statistically significantly larger for female than for male fetuses (*p* = 0.174); mean − 0.5 days for females (SD 2.7 days) and 0.2 days for males (SD 2.7 days), respectively.

The median maternal age of the women at first study visit was 29 years. Body mass index (BMI) at first antenatal visit covered a range of 16.7–44.8 kg/m^2^ , with a median BMI of 23.5 kg/m^2^. Fourteen women (2.4%) were underweight (BMI < 18.5 kg/m^2^), 145 (24.9%) were overweight (BMI 25.0–29.9 kg/m^2^), and 64 (11.0%) were obese (BMI > 30.0 kg/m^2^). The majority of the study population, 92%, were born in a Nordic country (Sweden, Norway, Denmark, Finland or Iceland), and 5.8% were of non-European origin. The median pregnancy duration at birth was 281 days.

Data on neonatal characteristics was available for 574 children (98.5%), with median birthweight of 3625 g and median birth length of 51 cm. The mean birthweight was 3645 g. For children with a mother born in a Nordic country, the mean birthweight was 3652 g, compared with 3563 g for children with a mother born in a non-Nordic country; a difference not statistically significant (*p* = 0.189)*.* The mean birthweight was neither significantly different for children with a mother born in a European compared with a non-European country; 3650 g versus 3563 g (*p* = 0.269). There was no statistically significant difference in mean birthweight between study sites; 3621 g in Uppsala, 3770 g in Falun, 3699 g in Katrineholm, 3606 g in Västerås, and 3591 g in Örebro, respectively (*p* = 0.051) Maternal and neonatal characteristics are summarized in Table 1.

**Table 1:** Maternal and neonatal characteristics.

Parameter	Median (IQR)	Range	n (%)
Maternal age (years)	29 (26, 33)	19, 44	
Maternal height (cm)	167 (163, 171)	148, 187	
Weight first visit (kg)	66 (60, 75)	43, 146	
Body mass index first visit (kg/m^2^)	23.5 (21,6, 26,5)	16.7, 44.8	
Nordic country of birth			537 (92.1%)
Non-European country of birth			34 (5.8%)
Smoking first visit			0
Nulliparous			250 (42.9%)
Gestational age at inclusion (days)	92 (90, 94)	87, 101	
Gestational age at delivery (days)	281 (276, 288)	259, 299	
Spontaneous vaginal delivery			458 (78.6%)
Induction of labour			40 (6.9%)
Newborn sex male			308 (52.8%)
APGAR < 7 at 5 min			6 (1.0%)
Neonatal death			0
Birthweight (g)	3625 (3344, 3925)	2366, 5100	
Birth length (cm)	51 (50, 52)	43, 57	

The EFW using Hadlock’s 3rd formula was compared with the actual birthweight in women who delivered within 2 days after the last ultrasound scan (n = 36). The difference between the mean EFW and actual birthweight was 13 g (*p* = 0.798).

The median and variance of EFW by GA for male and female fetuses from gestational week 12 until gestational week 42 are shown in Tables 2 and 3 and Eq. (1). The variance is expressed in standard deviations (+ 3 SD, + 2 SD, + 1 SD, median, − 1 SD, − 2 SD and − 3 SD) in Table 2 and in percentiles (2.5th, 5th, 10th, 25th, median, 75th, 90th, 95th and 97.5th) in Table 3.

**Table 2:** Medians and standard deviations (SD) of estimated fetal weight in grams by gestational age (GA) for male and female fetuses.

GA (weeks^a^)	− 3 SD	− 2 SD	− 1 SD	Median	+ 1 SD	+ 2 SD	+ 3 SD
12	45	48	50	53	56	59	63
13	59	62	66	69	74	78	82
14	75	80	85	90	95	101	107
15	95	101	108	115	122	129	138
16	120	127	136	145	154	164	175
17	149	159	169	181	193	206	220
18	183	196	210	224	240	257	275
19	223	240	257	276	296	317	340
20	270	291	312	336	361	388	417
21	324	350	377	406	437	471	508
22	386	417	451	487	526	568	613
23	457	495	535	579	627	678	734
24	537	582	631	684	741	804	872
25	626	679	738	802	871	946	1028
26	724	788	858	933	1016	1105	1203
27	832	907	989	1078	1176	1282	1398
28	950	1038	1133	1238	1352	1476	1612
29	1077	1178	1289	1410	1543	1688	1847
30	1212	1328	1456	1596	1749	1917	2101
31	1355	1487	1633	1793	1969	2163	2375
32	1503	1654	1819	2002	2202	2423	2666
33	1655	1825	2012	2218	2446	2697	2973
34	1810	2000	2210	2442	2698	2981	3294
35	1964	2175	2409	2669	2956	3275	3628
36	2114	2348	2608	2897	3218	3574	3970
37	2257	2515	2802	3123	3479	3876	4319
38	2391	2674	2989	3342	3736	4177	4670
39	2512	2819	3164	3552	3986	4474	5021
40	2616	2949	3324	3747	4224	4762	5367
41	2701	3060	3466	3926	4447	5037	5705
42	2764	3148	3585	4083	4649	5295	6030

^a^GA expressed as completed gestational weeks, e.g. 12 weeks corresponds to 12 + 0 weeks or 84 gestational days.

**Table 3:** Medians and percentiles of estimated fetal weight in grams by gestational age (GA) for male and female fetuses.

GA (weeks^a^)	2.5th	5th	10th	25th	Median	75th	90th	95th	97.5th
12	48	49	50	51	53	55	57	58	59
13	62	63	65	67	69	72	75	76	78
14	80	81	83	86	90	93	97	99	101
15	102	104	106	110	115	119	124	127	129
16	128	130	133	139	145	151	157	161	164
17	159	162	166	173	181	189	197	202	206
18	196	201	206	214	224	235	245	251	256
19	240	246	252	263	276	289	302	309	316
20	291	298	306	320	336	353	369	378	387
21	351	359	369	386	406	427	447	459	470
22	419	429	441	462	487	513	537	552	566
23	496	509	523	549	579	611	641	659	676
24	584	599	617	648	684	722	759	781	801
25	682	700	721	758	802	848	892	919	943
26	791	812	837	882	933	988	1040	1073	1102
27	911	936	965	1017	1078	1143	1205	1243	1277
28	1041	1071	1105	1166	1238	1313	1386	1431	1471
29	1182	1216	1257	1327	1410	1498	1583	1635	1682
30	1333	1372	1419	1500	1596	1698	1795	1856	1910
31	1493	1538	1591	1684	1793	1910	2022	2092	2154
32	1660	1711	1771	1877	2002	2135	2262	2342	2414
33	1832	1889	1958	2077	2218	2369	2514	2605	2686
34	2008	2072	2148	2283	2442	2612	2775	2878	2970
35	2184	2255	2341	2491	2669	2859	3043	3158	3262
36	2358	2437	2532	2699	2897	3110	3315	3444	3559
37	2526	2614	2718	2903	3123	3359	3587	3730	3860
38	2686	2782	2897	3100	3342	3603	3856	4015	4159
39	2832	2937	3063	3286	3552	3839	4118	4294	4453
40	2963	3077	3214	3457	3747	4062	4369	4563	4739
41	3075	3198	3346	3609	3926	4270	4606	4819	5011
42	3164	3297	3456	3740	4083	4456	4823	5056	5267

^a^GA expressed as completed gestational weeks, e.g. 12 weeks corresponds to 12 + 0 weeks or 84 gestational days.

Equation 1. Mean and variance for estimated fetal weight in male and female fetuses.

1 $$\begin{aligned} E(Z_{{\text{i}}} ) \, & = \, - {2}.{8}0 \, + \, \left[ {{1}.{\text{97 GA}}_{{\text{i}}}^{{0.{5}}} } \right] \, + \, \left[ { - 0.0000{\text{2 GA}}_{{\text{i}}}^{{3}} } \right] \\ Var(Z_{{\text{i}}} ) \, & = \, 0.0{2 } + \, \left[ { - 0.00{\text{2 GA}}_{{\text{i}}} } \right] \, + \, \left[ { - 0.0{\text{1 GA}}_{{\text{i}}}^{{0.{5}}} } \right] \, + \, \left[ {{2}.{\text{39e}} - 0{\text{7 GA}}_{{\text{i}}}^{{3}} } \right] \, \\ & \quad + \, \left[ { - {9}.{\text{92e}} - 0{\text{8 GA}}_{{\text{i}}}^{{0.{5}}} {\text{GA}}_{{\text{i}}}^{{3}} } \right] \, + \, \left[ {{4}.{\text{12e}} - {\text{12 GA}}_{{\text{i}}}^{{6}} } \right] \\ \end{aligned}$$

**Figure 2:**
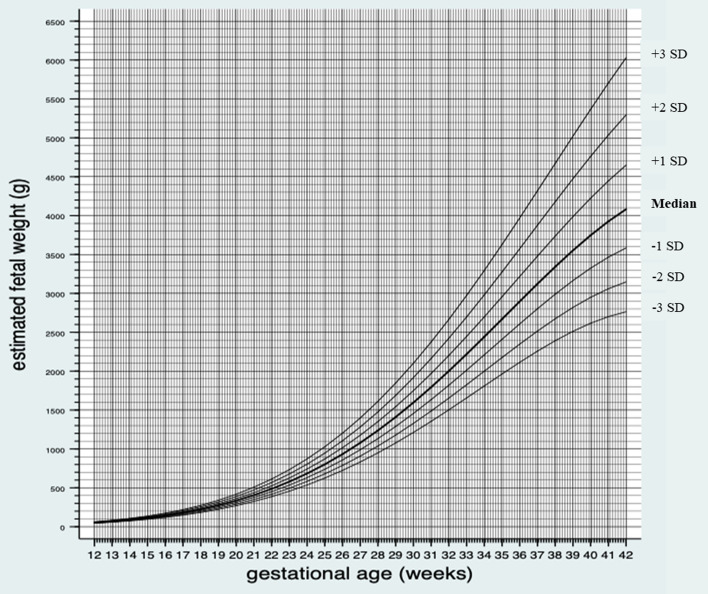
Median and variance in standard deviations (SD) (+ 3 SD, + 2 SD, + 1 SD, median, − 1 SD, − 2 SD and − 3 SD) of estimated fetal weight in grams (g) by gestational age for male and female fetuses.

Figure 2 shows the median and variance in SD of EFW by GA from gestational day 84 (gestational week 12 + 0) until gestational day 294 (gestational week 42 + 0). Supplementary table 1a shows the medians and variance in SD of EFW by GA for male and female fetuses for each day of the pregnancy from gestational day 84 until gestational day 294.

**Figure 3:**
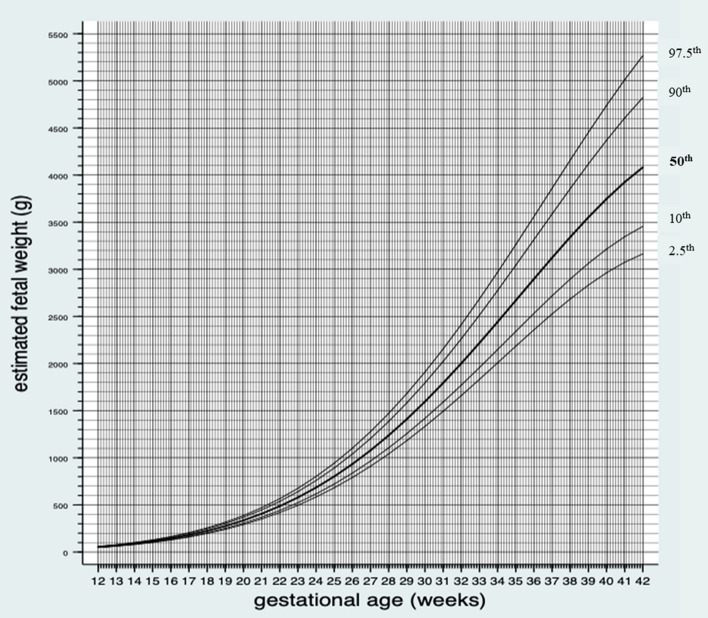
Median and variance in percentiles (2.5th, 10th, 50th, 90th and 97.5th) of estimated fetal weight by gestational age for male and female fetuses.

Figure 3 shows the median and variance in percentiles of EFW by GA from gestational day 84 until gestational day 294. Supplementary table 1b shows the medians and variance in percentiles of EFW by GA for male and female fetuses for each day of the pregnancy from gestational day 84 until gestational day 294.

**Figure 4:**
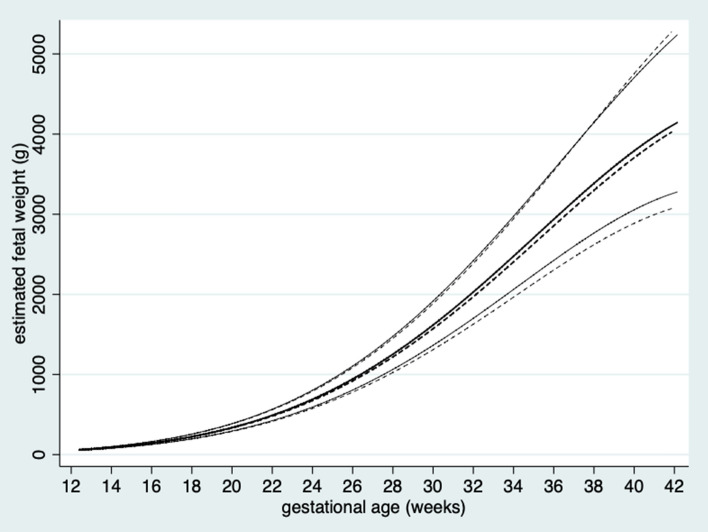
Sex specific reference ranges for estimated fetal weight by gestational age at 2.5th, 50th and 97.5th percentiles for male fetuses (continuous) and female fetuses (dashed).

Figure 4 shows the sex specific reference ranges for EFW by GA at 2.5th, 50th and 97.5th percentiles. Supplementary tables 2a-b show the medians and variance in SD (2a) and percentiles (2b) of EFW by GA for male fetuses. Supplementary tables 3a-b show the medians and variance in SD (3a) and percentiles (3b) of EFW by GA for female fetuses. All supplemental tables enclose the full equations of the median and variance for EFW.

**Figure 5:**
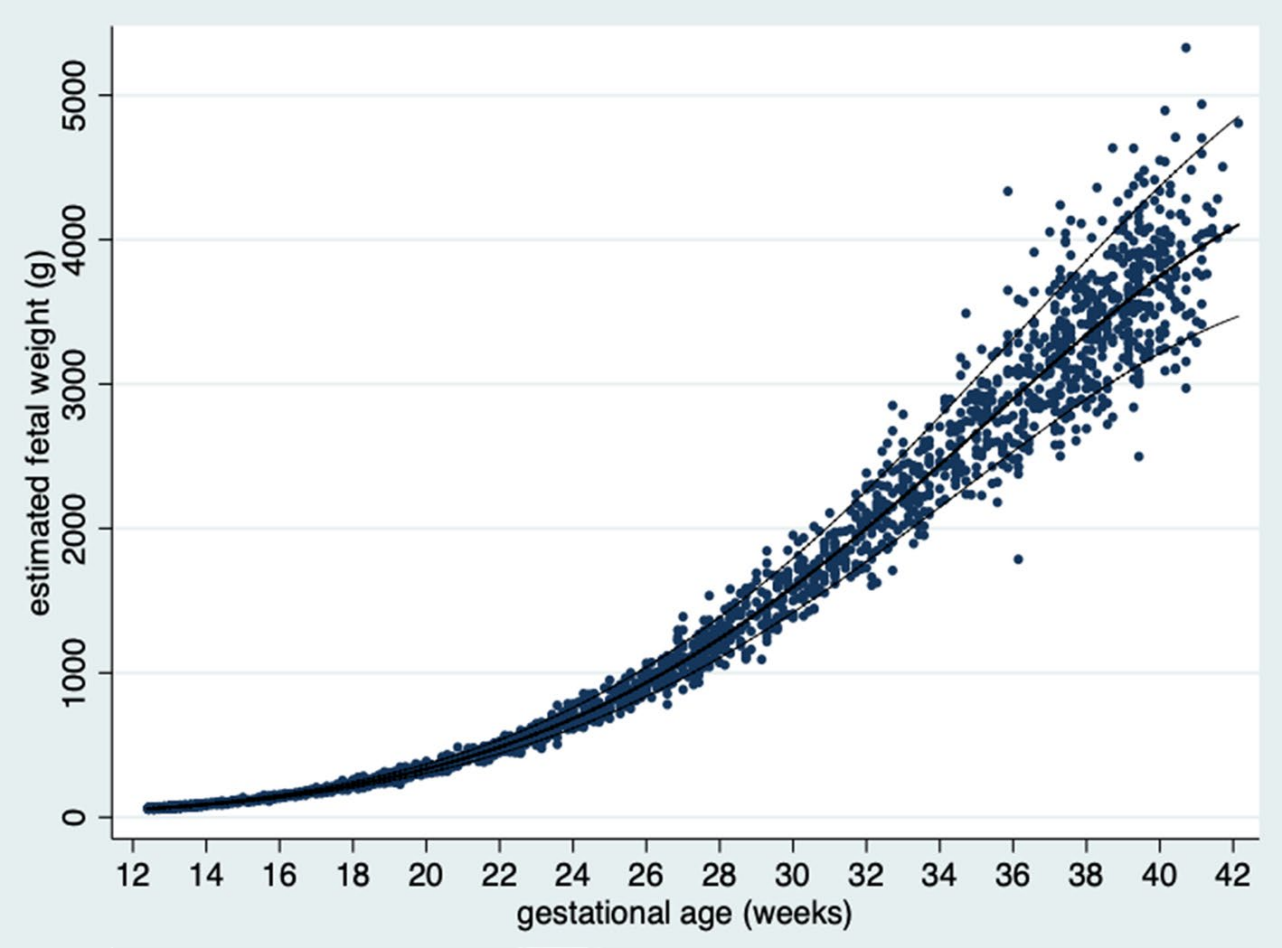
Raw data (n = 2551) with fitted percentiles (10th, 50th, 90th) for estimated fetal weight by gestational age.

Figure 5 shows the distribution of the raw data with fitted percentiles for estimated fetal weight by GA.

**Figure 6:**
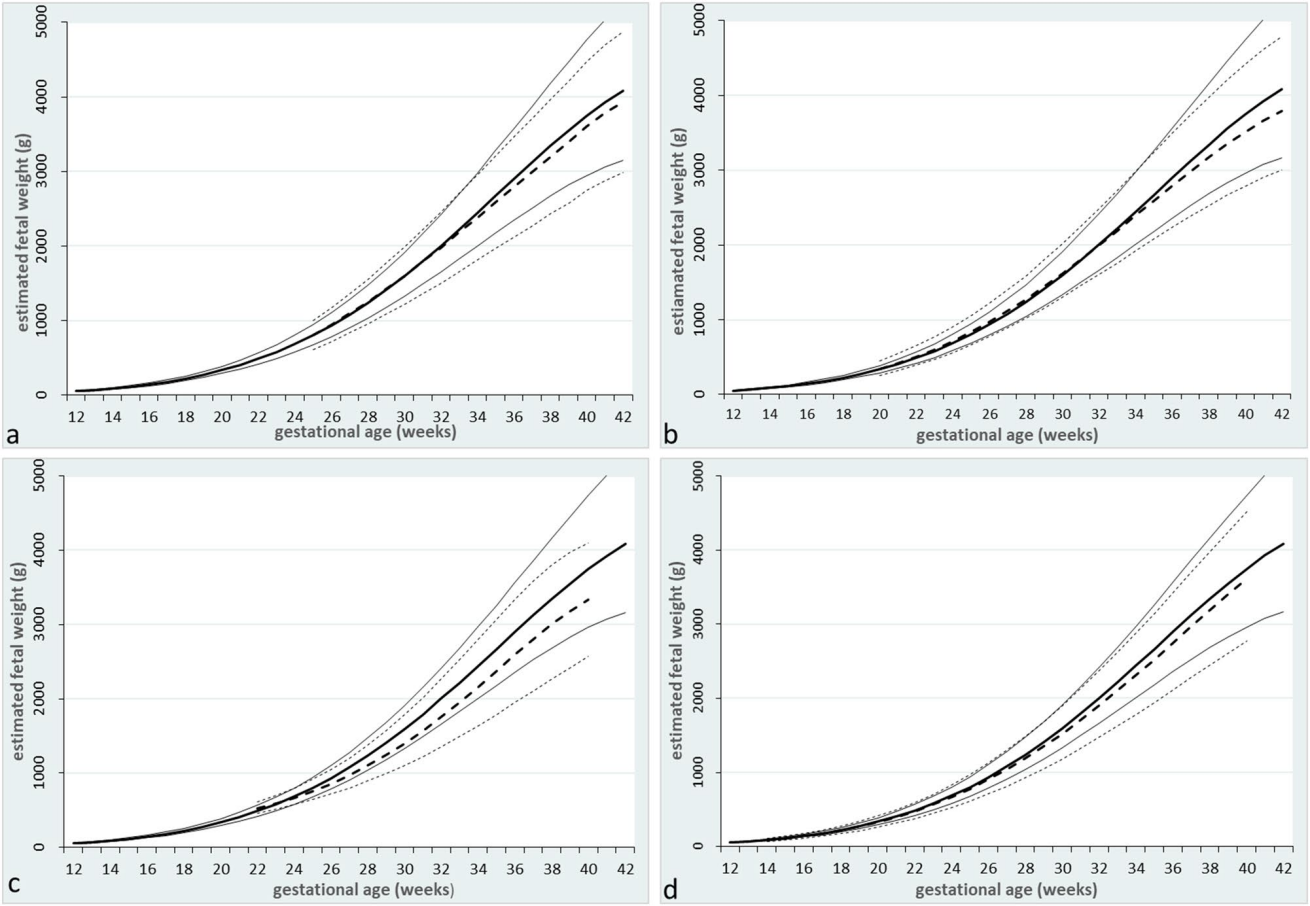
Comparisons of estimated fetal weight for males and females in the present study with other reference ranges; (**a**) new reference ranges (continuous) versus Maršál et al^10^ (dashed), + 2 SD, median, − 2 SD; (**b**) new reference ranges (continuous) versus Johnsen et al^36^ (dashed), 2.5th percentile, median, 97.5th percentile; (**c**) new reference ranges (continuous) versus INTERGROWTH-21st^21^ (dashed), 2.5th percentile, median, 97.5th percentile; (**d**) new reference ranges (continuous) versus WHO^20^ (dashed), 2.5th percentile, median, 97.5th percentile.

In comparison with the currently used Swedish growth reference ranges by Maršál et al^10^, the new reference ranges had comparable median EFW until gestational week 33, see Fig. 6a. Thereafter, the differences increased with advancing GA, with higher median EFW in the new reference ranges. From gestational week 25 to 33, the difference in median EFW was less than 2%. In week 39, the difference in median EFW was the largest; 4.4%, corresponding to 158 g. For + 2 SD of EFW, the difference exceeded 5% from week 38, and was the highest in week 42; 7.9%, corresponding to 419 g. Before gestational week 33, + 2 SD of EFW was lower in the new reference ranges. In the new reference ranges, − 2 SD corresponded to approximately 10% higher EFW from week 25 to 39, thereafter the differences declined, but − 2 SD of EFW remained higher in the new reference ranges throughout pregnancy. In week 38, the difference was 244 g, or 9.1%, and in week 41, 205 g, or 7.0%.

Next, the new reference ranges were compared with the Norwegian reference ranges by Johnsen et al. from 2006^36^, the standards from the INTERGROWTH-21st project^21^ and WHO^20^, see Fig. 6b–d. Compared with the Norwegian reference ranges, the new reference ranges had lower median EFW until week 31, and thereafter higher median EFW, see Fig. 6b. In the new reference ranges, the 2.5th percentile was higher in all gestational weeks, with the most pronounced differences in late pregnancy. Compared with the INTERGROWTH-21st project, the median, the 2.5th, and the 97.5th percentiles of EFW were higher in the new reference ranges from gestational week 24 and onwards, see Fig. 6c. The new reference ranges had higher median EFW also in comparison with WHO; 3.6–5.3% from gestational week 27 and forward. In the new reference ranges, the 2.5th percentile was higher throughout pregnancy, declining from 12.7% in week 15, to 10.4% in week 36, the later corresponding to 245 g. Thereafter, the difference was below 10%. The 97.5th percentile was lower in the new reference ranges in early pregnancy, but the curves crossed in week 29, with a maximum of 4.7%, or 224 g, in week 40.

When comparing women with BMI 18.5–29.9 kg/m^2^ in early pregnancy to the whole study population, there were no statistically significant differences in median EFW (*p* = 0.991 for both sexes, *p* = 0.985 for males and *p* = 0.998 for females). The difference in median EFW was the largest in week 42 (14 g). Supplementary table 4a-b show the medians and variance in SD (4a) and percentiles (4b) of EFW by GA for male and female fetuses in the subset of women with BMI 18.5–29.9 kg/m^2^.

## Discussion

In this study we present new Swedish reference ranges of EFW by GA to be used for assessing fetal weight and growth from gestational week 12 to 42.

A strength of the study is the adherence to a recommended study design for construction of reference ranges of fetal size and growth^7,8,37^. Large efforts have been made to ensure that the study population only includes pregnancies with low risk of aberrant fetal growth. Another strength is the prospective enrolment of study subjects and the use of modern statistical methods, which take into account the repeated measurements of each fetus and increased variation of EFW with advancing GA. The study design and statistical methods allow the growth reference ranges to be used to reliably evaluate EFW, both regarding size and growth. Moreover, the scans were performed by a limited number of experienced sonographers, and the quality of the original data was high, with a low rate of erroneous data^29^.

Another major strength of the study is the distribution of ultrasound scans throughout the entire second and third trimester. This is particularly valuable in comparison with the currently used Swedish growth reference ranges by Maršál et al., which do not include any intrauterine measurements before gestational week 25^10^. Assessment of EFW is an important part of obstetric care, even before the limit of extrauterine viability, practically considered as gestational week 22 + 0 in Sweden today. If the new reference ranges are adopted in clinical practice, assessment of EFW and postnatal growth in the extreme preterm period will be compared with reference ranges based on observational data rather than the currently used extrapolated data.

A limitation of the study is the homogeneity of the study population. In order to recruit a study cohort with similar ethnic background as the Swedish pregnant population, the primary care centres that recruited study subjects were singled out with great care. Several of the centres were situated in areas with a large immigrated population. Further efforts were made to recruit study subjects of non-Nordic origin by offering study information not only in Swedish, but also in English and Arabic. However, less than 10% of the study subjects were born outside the Nordic countries, compared with 28.5% among Swedish pregnant women 2018^38^. The sampling bias towards homogenous ethnicity was probably unavoidable in part due to language difficulties, as interpreters could not be offered at all study visits due to limited financial means. Additionally, a higher percentage of the women born outside the Nordic countries were excluded from the study due to pregnancy complications; 15 versus 10%.

We included women regardless of their BMI in early pregnancy, despite the increased risks of restricted as well as exaggerated fetal growth associated with low and high BMI. The rationale behind this decision was that we aimed to recruit a study population similar to the current healthy pregnant population in Sweden. Mean maternal age and BMI were similar to those in the current Swedish pregnant population, and thus the study population can be seen as representative for Swedish pregnant women^38^. The vast majority of Swedish women of fertile age are well nourished, and low BMI is rarely a sign of undernutrition. However, the inclusion of women with obesity might affect the results in a way that skews the growth reference ranges towards a higher EFW, which might lead to an underestimation of the number of fetuses suspected as large for gestational age (LGA) during pregnancy. In that aspect, the inclusion of obese women is a limitation. The increased risk of pregnancy complications in women with increased BMI is reflected in the higher rate of exclusion due to pregnancy complications in overweight and obese women. However, in a sensitivity analysis, the growth reference ranges were compared with and without the inclusion of women with BMI less than 18.5 kg/m^2^ or more than 30 kg/m^2^. The difference in median EFW, as well as for − 2 SD and + 2 SD, was clinically insignificant if women with abnormal BMI were excluded. Therefore, we suggest that the results from the complete study population should be used, since the larger cohort is a strength in later GAs, as the variation in fetal weight increases and is better evaluated with a larger cohort. Moreover, the inclusion of women regardless of BMI entails a higher generalisability to the background population.

The mean birthweight in this study was approximately 150 g higher than the mean birthweight for all children born in Sweden in 2018; 3646 versus 3497 g^38^. In contrast to national data, the study cohort only includes children born term and of women without conditions that affect fetal growth. The mean birthweight can therefore be regarded as representative for Swedish term born children.

In clinical practice, there is a large variety of formulae used for estimating fetal weight. In this study, we used Hadlock et al.’s third formula for EFW, which includes the biometric measurements BPD, HC, AC and FL. The formula for EFW was chosen based on compelling evidence from repeated studies which have proven Hadlock et al.’s formulae to be superior or non-inferior to a variety of frequently used or more recently developed EFW formulae^15–18,39,40^. In Sweden, the formula adapted by Persson and Weldner in 1986 is recommended by the Swedish Society of Obstetrics and Gynecology and commonly used for estimation of fetal weight^30,41^. Since the Persson Weldner formula was not included in any of the cited studies, its accuracy is not evaluated in comparison with other formulae for EFW. Moreover, the accuracy of the Person Weldner formula has not been assessed in early gestational weeks. The different formulae presented by Hadlock et al. include various biometric measurements, but their performance is largely comparable whether BPD is included or not^15^. In the study, we have only estimated fetal weight if HC and BPD were present. The use of Hadlock’s third formula in our study seems appropriate, since the difference between mean EFW and actual birthweight was negligible in women who delivered within two days from the last ultrasound scan.

Over the years, a multitude of national and international standards of EFW have been presented. Despite the large efforts made during the last decade to create a standard that can be universally adopted, doubts have been raised regarding the notion of “one size fits all”^20,22–27^. Vieira et al. compared the proportion of children classified as small for gestational age (SGA) and LGA at birth using three different growth reference ranges in a Swedish cohort^42^. The standard presented by the INTERGROWTH-21st project only classified 3.1% as below the 10th percentile for GA and sex. In concordance with these findings, the difficulty in implementing international standards have repeatedly been highlighted since the sensitivity and specificity in detecting growth restricted fetuses or fetuses with adverse perinatal outcome vary widely in different populations^12,43–46^. Thus, great care must be taken in choosing a national reference for fetal size and growth.

Compared with the currently used Swedish growth reference ranges by Maršál et al., the EFW was considerably higher in our study, especially in late pregnancy. The higher EFW in our study might in part be explained by the stricter inclusion criteria, e.g. women who smoked and had other risk factors for fetal growth restriction were not eligible. Further, there were large differences between our and Maršál et al.’s reference ranges at all GAs for the EFW at − 2 SD, which is used as cut-off for SGA in Sweden. The larger span in late GAs between + 2 and − 2 SD in our reference ranges may be a reflection of how a larger study sample and modern statistical methods estimate the increasing variation in fetal weight with increased GA in a correct way.

The pregnant populations in the Nordic countries are quite homogeneous, with similar mean birthweight for live born children, ranging from 3484 g in Denmark to 3501 g in Sweden, and the somewhat higher Icelandic mean of 3592 g^47^. We compared our reference ranges with the most recently published Nordic reference ranges by Johnsen et al^36^. The higher median EFW in our reference ranges is most likely explained by the fact that Johnsen et al. did not exclude women with pregnancy complications from the analyses, rather than by differences in the populations. The reference ranges created by INTERGROWTH-21st project and the WHO on the other hand are based on populations that are quite different from the Swedish pregnant population and our study population, with shorter maternal height (162 cm vs 167 cm), higher rate of nulliparous study subjects (68% vs 43%) and lower mean birthweight (3.3 kg vs 3.6 kg). We believe that optimal fetal weight is underestimated if these international reference ranges are used in the Nordic populations, but further studies are needed to confirm this speculation.

The distribution of percentiles in a growth reference range will affect the percentage of fetuses that are considered SGA or LGA. Hence, if the birthweight percentiles in the population are significantly higher than the percentiles of the reference range used, SGA fetuses are expected to be misclassified as appropriate for GA. Accordingly, using the presently used reference ranges, their increased risk of adverse perinatal outcome is most probably overseen. Likewise, if the mean birthweight is lower than the 50th percentile of the reference range, an increased number of fetuses are falsely suspected as SGA, and thereby risk unnecessary obstetric interventions with increased risk of adverse perinatal outcome^48^. If these new reference ranges with higher EFW percentiles during the third trimester are adopted in clinical practice, the risk of missing to identify growth restricted fetuses might become lower in comparison to using the current growth reference ranges or standards created by INTERGROWTH-21st project or the WHO. Likewise, the risk of misclassifying fetuses as LGA might be lower with our new reference ranges, and hence the risk of unnecessary interventions should be reduced. Further studies are needed to evaluate the sensitivity and specificity of the new growth reference ranges to detect SGA fetuses, and to assess appropriate cut-offs for SGA and LGA in order to identify fetuses at high risk of adverse perinatal outcome, both from an obstetric and neonatal perspective.

## Supplementary information

Supplementary Tables.

## Data Availability

The datasets generated during and/or analyzed during the current study are not publicly available due to the ethical and legal restrictions prohibiting the sharing of personal data, but are available from the corresponding author on reasonable request.
