# Supplementary material for: Swedish intrauterine growth reference ranges for estimated fetal weight
Source: Sci Rep. 2021 Jun 14;11:12464. doi: 10.1038/s41598-021-92032-2 (PMC8203766; doi:10.1038/s41598-021-92032-2)
Supplement: Supplementary file 1 — Supplementary Tables. [file 41598_2021_92032_MOESM1_ESM.pdf]

Supplemental table 1a. Medians and standard deviations (SD) of estimated fetal weight in grams by gestational age (GA) in days for male and female fetuses.

GA (days)*	-3 SD	-2 SD	-1 SD	Median	+1 SD	+2 SD	+3 SD
84	45	48	50	53	56	59	63
85	45	48	50	53	56	59	63
86	45	48	50	53	56	59	63
87	45	48	50	53	56	59	63
88	45	48	50	53	56	59	63
89	45	48	50	53	56	59	63
90	45	48	50	53	56	59	63
91	59	62	66	69	74	78	82
92	59	62	66	69	74	78	82
93	59	62	66	69	74	78	82
94	59	62	66	69	74	78	82
95	59	62	66	69	74	78	82
96	59	62	66	69	74	78	82
97	59	62	66	69	74	78	82
98	75	80	85	90	95	101	107
99	75	80	85	90	95	101	107
100	75	80	85	90	95	101	107
101	75	80	85	90	95	101	107
102	75	80	85	90	95	101	107
103	75	80	85	90	95	101	107
104	75	80	85	90	95	101	107
105	95	101	108	115	122	129	138
106	95	101	108	115	122	129	138
107	95	101	108	115	122	129	138
108	95	101	108	115	122	129	138
109	95	101	108	115	122	129	138
110	95	101	108	115	122	129	138
111	95	101	108	115	122	129	138
112	120	127	136	145	154	164	175
113	120	127	136	145	154	164	175
114	120	127	136	145	154	164	175
115	120	127	136	145	154	164	175
116	120	127	136	145	154	164	175
117	120	127	136	145	154	164	175
118	120	127	136	145	154	164	175
119	149	159	169	181	193	206	220
120	149	159	169	181	193	206	220
121	149	159	169	181	193	206	220
122	149	159	169	181	193	206	220
123	149	159	169	181	193	206	220
124	149	159	169	181	193	206	220
125	149	159	169	181	193	206	220
126	183	196	210	224	240	257	275
127	183	196	210	224	240	257	275
128	183	196	210	224	240	257	275
129	183	196	210	224	240	257	275

130	183	196	210	224	240	257	275
131	183	196	210	224	240	257	275
132	183	196	210	224	240	257	275
133	223	240	257	276	296	317	340
134	223	240	257	276	296	317	340
135	223	240	257	276	296	317	340
136	223	240	257	276	296	317	340
137	223	240	257	276	296	317	340
138	223	240	257	276	296	317	340
139	223	240	257	276	296	317	340
140	270	291	312	336	361	388	417
141	270	291	312	336	361	388	417
142	270	291	312	336	361	388	417
143	270	291	312	336	361	388	417
144	270	291	312	336	361	388	417
145	270	291	312	336	361	388	417
146	270	291	312	336	361	388	417
147	324	350	377	406	437	471	508
148	324	350	377	406	437	471	508
149	324	350	377	406	437	471	508
150	324	350	377	406	437	471	508
151	324	350	377	406	437	471	508
152	324	350	377	406	437	471	508
153	324	350	377	406	437	471	508
154	386	417	451	487	526	568	613
155	386	417	451	487	526	568	613
156	386	417	451	487	526	568	613
157	386	417	451	487	526	568	613
158	386	417	451	487	526	568	613
159	386	417	451	487	526	568	613
160	386	417	451	487	526	568	613
161	457	495	535	579	627	678	734
162	457	495	535	579	627	678	734
163	457	495	535	579	627	678	734
164	457	495	535	579	627	678	734
165	457	495	535	579	627	678	734
166	457	495	535	579	627	678	734
167	457	495	535	579	627	678	734
168	537	582	631	684	741	804	872
169	537	582	631	684	741	804	872
170	537	582	631	684	741	804	872
171	537	582	631	684	741	804	872
172	537	582	631	684	741	804	872
173	537	582	631	684	741	804	872
174	537	582	631	684	741	804	872
175	626	679	738	802	871	946	1028
176	626	679	738	802	871	946	1028
177	626	679	738	802	871	946	1028

178	626	679	738	802	871	946	1028
179	626	679	738	802	871	946	1028
180	626	679	738	802	871	946	1028
181	626	679	738	802	871	946	1028
182	724	788	858	933	1016	1105	1203
183	724	788	858	933	1016	1105	1203
184	724	788	858	933	1016	1105	1203
185	724	788	858	933	1016	1105	1203
186	724	788	858	933	1016	1105	1203
187	724	788	858	933	1016	1105	1203
188	724	788	858	933	1016	1105	1203
189	832	907	989	1078	1176	1282	1398
190	832	907	989	1078	1176	1282	1398
191	832	907	989	1078	1176	1282	1398
192	832	907	989	1078	1176	1282	1398
193	832	907	989	1078	1176	1282	1398
194	832	907	989	1078	1176	1282	1398
195	832	907	989	1078	1176	1282	1398
196	950	1038	1133	1238	1352	1476	1612
197	950	1038	1133	1238	1352	1476	1612
198	950	1038	1133	1238	1352	1476	1612
199	950	1038	1133	1238	1352	1476	1612
200	950	1038	1133	1238	1352	1476	1612
201	950	1038	1133	1238	1352	1476	1612
202	950	1038	1133	1238	1352	1476	1612
203	1077	1178	1289	1410	1543	1688	1847
204	1077	1178	1289	1410	1543	1688	1847
205	1077	1178	1289	1410	1543	1688	1847
206	1077	1178	1289	1410	1543	1688	1847
207	1077	1178	1289	1410	1543	1688	1847
208	1077	1178	1289	1410	1543	1688	1847
209	1077	1178	1289	1410	1543	1688	1847
210	1212	1328	1456	1596	1749	1917	2101
211	1212	1328	1456	1596	1749	1917	2101
212	1212	1328	1456	1596	1749	1917	2101
213	1212	1328	1456	1596	1749	1917	2101
214	1212	1328	1456	1596	1749	1917	2101
215	1212	1328	1456	1596	1749	1917	2101
216	1212	1328	1456	1596	1749	1917	2101
217	1355	1487	1633	1793	1969	2163	2375
218	1355	1487	1633	1793	1969	2163	2375
219	1355	1487	1633	1793	1969	2163	2375
220	1355	1487	1633	1793	1969	2163	2375
221	1355	1487	1633	1793	1969	2163	2375
222	1355	1487	1633	1793	1969	2163	2375
223	1355	1487	1633	1793	1969	2163	2375
224	1503	1654	1819	2002	2202	2423	2666
225	1503	1654	1819	2002	2202	2423	2666

226	1503	1654	1819	2002	2202	2423	2666
227	1503	1654	1819	2002	2202	2423	2666
228	1503	1654	1819	2002	2202	2423	2666
229	1503	1654	1819	2002	2202	2423	2666
230	1503	1654	1819	2002	2202	2423	2666
231	1655	1825	2012	2218	2446	2697	2973
232	1655	1825	2012	2218	2446	2697	2973
233	1655	1825	2012	2218	2446	2697	2973
234	1655	1825	2012	2218	2446	2697	2973
235	1655	1825	2012	2218	2446	2697	2973
236	1655	1825	2012	2218	2446	2697	2973
237	1655	1825	2012	2218	2446	2697	2973
238	1810	2000	2210	2442	2698	2981	3294
239	1810	2000	2210	2442	2698	2981	3294
240	1810	2000	2210	2442	2698	2981	3294
241	1810	2000	2210	2442	2698	2981	3294
242	1810	2000	2210	2442	2698	2981	3294
243	1810	2000	2210	2442	2698	2981	3294
244	1810	2000	2210	2442	2698	2981	3294
245	1964	2175	2409	2669	2956	3275	3628
246	1964	2175	2409	2669	2956	3275	3628
247	1964	2175	2409	2669	2956	3275	3628
248	1964	2175	2409	2669	2956	3275	3628
249	1964	2175	2409	2669	2956	3275	3628
250	1964	2175	2409	2669	2956	3275	3628
251	1964	2175	2409	2669	2956	3275	3628
252	2114	2348	2608	2897	3218	3574	3970
253	2114	2348	2608	2897	3218	3574	3970
254	2114	2348	2608	2897	3218	3574	3970
255	2114	2348	2608	2897	3218	3574	3970
256	2114	2348	2608	2897	3218	3574	3970
257	2114	2348	2608	2897	3218	3574	3970
258	2114	2348	2608	2897	3218	3574	3970
259	2257	2515	2802	3123	3479	3876	4319
260	2257	2515	2802	3123	3479	3876	4319
261	2257	2515	2802	3123	3479	3876	4319
262	2257	2515	2802	3123	3479	3876	4319
263	2257	2515	2802	3123	3479	3876	4319
264	2257	2515	2802	3123	3479	3876	4319
265	2257	2515	2802	3123	3479	3876	4319
266	2391	2674	2989	3342	3736	4177	4670
267	2391	2674	2989	3342	3736	4177	4670
268	2391	2674	2989	3342	3736	4177	4670
269	2391	2674	2989	3342	3736	4177	4670
270	2391	2674	2989	3342	3736	4177	4670
271	2391	2674	2989	3342	3736	4177	4670
272	2391	2674	2989	3342	3736	4177	4670
273	2512	2819	3164	3552	3986	4474	5021

274	2512	2819	3164	3552	3986	4474	5021
275	2512	2819	3164	3552	3986	4474	5021
276	2512	2819	3164	3552	3986	4474	5021
277	2512	2819	3164	3552	3986	4474	5021
278	2512	2819	3164	3552	3986	4474	5021
279	2512	2819	3164	3552	3986	4474	5021
280	2616	2949	3324	3747	4224	4762	5367
281	2616	2949	3324	3747	4224	4762	5367
282	2616	2949	3324	3747	4224	4762	5367
283	2616	2949	3324	3747	4224	4762	5367
284	2616	2949	3324	3747	4224	4762	5367
285	2616	2949	3324	3747	4224	4762	5367
286	2616	2949	3324	3747	4224	4762	5367
287	2701	3060	3466	3926	4447	5037	5705
288	2701	3060	3466	3926	4447	5037	5705
289	2701	3060	3466	3926	4447	5037	5705
290	2701	3060	3466	3926	4447	5037	5705
291	2701	3060	3466	3926	4447	5037	5705
292	2701	3060	3466	3926	4447	5037	5705
293	2701	3060	3466	3926	4447	5037	5705
294	2764	3148	3585	4083	4649	5295	6030

Mean and variance equation for male and female fetuses:

$E(Z_i) = -2.796656251349332 + [1.965246568058469\ GA_i^{0.5}] + [-0.000021935076611\ GA_i^{3}]$

$Var(Z_i) = 0.0191773432342972 + [0.0017826895572236\ GA_i] + [-0.01077702329383\ GA_i^{0.5}] + [2.38595060716e\text{-}07\ GA_i^{3}] + [-9.91625483550e\text{-}08\ GA_i^{0.5} GA_i^{3}] + [4.12225895831e\text{-}12\ GA_i^{6}]$

Supplemental table 1b. Medians and percentiles of estimated fetal weight in grams by gestational age (GA) in days for male and female fetuses.

GA (days)	2.5th	5th	10th	25th	Median	75th	90th	95th	97.5th
84	48	49	50	51	53	55	57	58	59
85	48	49	50	51	53	55	57	58	59
86	48	49	50	51	53	55	57	58	59
87	48	49	50	51	53	55	57	58	59
88	48	49	50	51	53	55	57	58	59
89	48	49	50	51	53	55	57	58	59
90	48	49	50	51	53	55	57	58	59
91	62	63	65	67	69	72	75	76	78
92	62	63	65	67	69	72	75	76	78
93	62	63	65	67	69	72	75	76	78
94	62	63	65	67	69	72	75	76	78
95	62	63	65	67	69	72	75	76	78
96	62	63	65	67	69	72	75	76	78
97	62	63	65	67	69	72	75	76	78
98	80	81	83	86	90	93	97	99	101
99	80	81	83	86	90	93	97	99	101
100	80	81	83	86	90	93	97	99	101
101	80	81	83	86	90	93	97	99	101
102	80	81	83	86	90	93	97	99	101
103	80	81	83	86	90	93	97	99	101
104	80	81	83	86	90	93	97	99	101
105	102	104	106	110	115	119	124	127	129
106	102	104	106	110	115	119	124	127	129
107	102	104	106	110	115	119	124	127	129
108	102	104	106	110	115	119	124	127	129
109	102	104	106	110	115	119	124	127	129
110	102	104	106	110	115	119	124	127	129
111	102	104	106	110	115	119	124	127	129
112	128	130	133	139	145	151	157	161	164
113	128	130	133	139	145	151	157	161	164
114	128	130	133	139	145	151	157	161	164
115	128	130	133	139	145	151	157	161	164
116	128	130	133	139	145	151	157	161	164
117	128	130	133	139	145	151	157	161	164
118	128	130	133	139	145	151	157	161	164
119	159	162	166	173	181	189	197	202	206
120	159	162	166	173	181	189	197	202	206
121	159	162	166	173	181	189	197	202	206
122	159	162	166	173	181	189	197	202	206
123	159	162	166	173	181	189	197	202	206
124	159	162	166	173	181	189	197	202	206
125	159	162	166	173	181	189	197	202	206

126	196	201	206	214	224	235	245	251	256
127	196	201	206	214	224	235	245	251	256
128	196	201	206	214	224	235	245	251	256
129	196	201	206	214	224	235	245	251	256
130	196	201	206	214	224	235	245	251	256
131	196	201	206	214	224	235	245	251	256
132	196	201	206	214	224	235	245	251	256
133	240	246	252	263	276	289	302	309	316
134	240	246	252	263	276	289	302	309	316
135	240	246	252	263	276	289	302	309	316
136	240	246	252	263	276	289	302	309	316
137	240	246	252	263	276	289	302	309	316
138	240	246	252	263	276	289	302	309	316
139	240	246	252	263	276	289	302	309	316
140	291	298	306	320	336	353	369	378	387
141	291	298	306	320	336	353	369	378	387
142	291	298	306	320	336	353	369	378	387
143	291	298	306	320	336	353	369	378	387
144	291	298	306	320	336	353	369	378	387
145	291	298	306	320	336	353	369	378	387
146	291	298	306	320	336	353	369	378	387
147	351	359	369	386	406	427	447	459	470
148	351	359	369	386	406	427	447	459	470
149	351	359	369	386	406	427	447	459	470
150	351	359	369	386	406	427	447	459	470
151	351	359	369	386	406	427	447	459	470
152	351	359	369	386	406	427	447	459	470
153	351	359	369	386	406	427	447	459	470
154	419	429	441	462	487	513	537	552	566
155	419	429	441	462	487	513	537	552	566
156	419	429	441	462	487	513	537	552	566
157	419	429	441	462	487	513	537	552	566
158	419	429	441	462	487	513	537	552	566
159	419	429	441	462	487	513	537	552	566
160	419	429	441	462	487	513	537	552	566
161	496	509	523	549	579	611	641	659	676
162	496	509	523	549	579	611	641	659	676
163	496	509	523	549	579	611	641	659	676
164	496	509	523	549	579	611	641	659	676
165	496	509	523	549	579	611	641	659	676
166	496	509	523	549	579	611	641	659	676
167	496	509	523	549	579	611	641	659	676
168	584	599	617	648	684	722	759	781	801
169	584	599	617	648	684	722	759	781	801
170	584	599	617	648	684	722	759	781	801
171	584	599	617	648	684	722	759	781	801
172	584	599	617	648	684	722	759	781	801
173	584	599	617	648	684	722	759	781	801

174	584	599	617	648	684	722	759	781	801
175	682	700	721	758	802	848	892	919	943
176	682	700	721	758	802	848	892	919	943
177	682	700	721	758	802	848	892	919	943
178	682	700	721	758	802	848	892	919	943
179	682	700	721	758	802	848	892	919	943
180	682	700	721	758	802	848	892	919	943
181	682	700	721	758	802	848	892	919	943
182	791	812	837	882	933	988	1040	1073	1102
183	791	812	837	882	933	988	1040	1073	1102
184	791	812	837	882	933	988	1040	1073	1102
185	791	812	837	882	933	988	1040	1073	1102
186	791	812	837	882	933	988	1040	1073	1102
187	791	812	837	882	933	988	1040	1073	1102
188	791	812	837	882	933	988	1040	1073	1102
189	911	936	965	1017	1078	1143	1205	1243	1277
190	911	936	965	1017	1078	1143	1205	1243	1277
191	911	936	965	1017	1078	1143	1205	1243	1277
192	911	936	965	1017	1078	1143	1205	1243	1277
193	911	936	965	1017	1078	1143	1205	1243	1277
194	911	936	965	1017	1078	1143	1205	1243	1277
195	911	936	965	1017	1078	1143	1205	1243	1277
196	1041	1071	1105	1166	1238	1313	1386	1431	1471
197	1041	1071	1105	1166	1238	1313	1386	1431	1471
198	1041	1071	1105	1166	1238	1313	1386	1431	1471
199	1041	1071	1105	1166	1238	1313	1386	1431	1471
200	1041	1071	1105	1166	1238	1313	1386	1431	1471
201	1041	1071	1105	1166	1238	1313	1386	1431	1471
202	1041	1071	1105	1166	1238	1313	1386	1431	1471
203	1182	1216	1257	1327	1410	1498	1583	1635	1682
204	1182	1216	1257	1327	1410	1498	1583	1635	1682
205	1182	1216	1257	1327	1410	1498	1583	1635	1682
206	1182	1216	1257	1327	1410	1498	1583	1635	1682
207	1182	1216	1257	1327	1410	1498	1583	1635	1682
208	1182	1216	1257	1327	1410	1498	1583	1635	1682
209	1182	1216	1257	1327	1410	1498	1583	1635	1682
210	1333	1372	1419	1500	1596	1698	1795	1856	1910
211	1333	1372	1419	1500	1596	1698	1795	1856	1910
212	1333	1372	1419	1500	1596	1698	1795	1856	1910
213	1333	1372	1419	1500	1596	1698	1795	1856	1910
214	1333	1372	1419	1500	1596	1698	1795	1856	1910
215	1333	1372	1419	1500	1596	1698	1795	1856	1910
216	1333	1372	1419	1500	1596	1698	1795	1856	1910
217	1493	1538	1591	1684	1793	1910	2022	2092	2154
218	1493	1538	1591	1684	1793	1910	2022	2092	2154
219	1493	1538	1591	1684	1793	1910	2022	2092	2154
220	1493	1538	1591	1684	1793	1910	2022	2092	2154
221	1493	1538	1591	1684	1793	1910	2022	2092	2154

222	1493	1538	1591	1684	1793	1910	2022	2092	2154
223	1493	1538	1591	1684	1793	1910	2022	2092	2154
224	1660	1711	1771	1877	2002	2135	2262	2342	2414
225	1660	1711	1771	1877	2002	2135	2262	2342	2414
226	1660	1711	1771	1877	2002	2135	2262	2342	2414
227	1660	1711	1771	1877	2002	2135	2262	2342	2414
228	1660	1711	1771	1877	2002	2135	2262	2342	2414
229	1660	1711	1771	1877	2002	2135	2262	2342	2414
230	1660	1711	1771	1877	2002	2135	2262	2342	2414
231	1832	1889	1958	2077	2218	2369	2514	2605	2686
232	1832	1889	1958	2077	2218	2369	2514	2605	2686
233	1832	1889	1958	2077	2218	2369	2514	2605	2686
234	1832	1889	1958	2077	2218	2369	2514	2605	2686
235	1832	1889	1958	2077	2218	2369	2514	2605	2686
236	1832	1889	1958	2077	2218	2369	2514	2605	2686
237	1832	1889	1958	2077	2218	2369	2514	2605	2686
238	2008	2072	2148	2283	2442	2612	2775	2878	2970
239	2008	2072	2148	2283	2442	2612	2775	2878	2970
240	2008	2072	2148	2283	2442	2612	2775	2878	2970
241	2008	2072	2148	2283	2442	2612	2775	2878	2970
242	2008	2072	2148	2283	2442	2612	2775	2878	2970
243	2008	2072	2148	2283	2442	2612	2775	2878	2970
244	2008	2072	2148	2283	2442	2612	2775	2878	2970
245	2184	2255	2341	2491	2669	2859	3043	3158	3262
246	2184	2255	2341	2491	2669	2859	3043	3158	3262
247	2184	2255	2341	2491	2669	2859	3043	3158	3262
248	2184	2255	2341	2491	2669	2859	3043	3158	3262
249	2184	2255	2341	2491	2669	2859	3043	3158	3262
250	2184	2255	2341	2491	2669	2859	3043	3158	3262
251	2184	2255	2341	2491	2669	2859	3043	3158	3262
252	2358	2437	2532	2699	2897	3110	3315	3444	3559
253	2358	2437	2532	2699	2897	3110	3315	3444	3559
254	2358	2437	2532	2699	2897	3110	3315	3444	3559
255	2358	2437	2532	2699	2897	3110	3315	3444	3559
256	2358	2437	2532	2699	2897	3110	3315	3444	3559
257	2358	2437	2532	2699	2897	3110	3315	3444	3559
258	2358	2437	2532	2699	2897	3110	3315	3444	3559
259	2526	2614	2718	2903	3123	3359	3587	3730	3860
260	2526	2614	2718	2903	3123	3359	3587	3730	3860
261	2526	2614	2718	2903	3123	3359	3587	3730	3860
262	2526	2614	2718	2903	3123	3359	3587	3730	3860
263	2526	2614	2718	2903	3123	3359	3587	3730	3860
264	2526	2614	2718	2903	3123	3359	3587	3730	3860
265	2526	2614	2718	2903	3123	3359	3587	3730	3860
266	2686	2782	2897	3100	3342	3603	3856	4015	4159
267	2686	2782	2897	3100	3342	3603	3856	4015	4159
268	2686	2782	2897	3100	3342	3603	3856	4015	4159
269	2686	2782	2897	3100	3342	3603	3856	4015	4159

270	2686	2782	2897	3100	3342	3603	3856	4015	4159
271	2686	2782	2897	3100	3342	3603	3856	4015	4159
272	2686	2782	2897	3100	3342	3603	3856	4015	4159
273	2832	2937	3063	3286	3552	3839	4118	4294	4453
274	2832	2937	3063	3286	3552	3839	4118	4294	4453
275	2832	2937	3063	3286	3552	3839	4118	4294	4453
276	2832	2937	3063	3286	3552	3839	4118	4294	4453
277	2832	2937	3063	3286	3552	3839	4118	4294	4453
278	2832	2937	3063	3286	3552	3839	4118	4294	4453
279	2832	2937	3063	3286	3552	3839	4118	4294	4453
280	2963	3077	3214	3457	3747	4062	4369	4563	4739
281	2963	3077	3214	3457	3747	4062	4369	4563	4739
282	2963	3077	3214	3457	3747	4062	4369	4563	4739
283	2963	3077	3214	3457	3747	4062	4369	4563	4739
284	2963	3077	3214	3457	3747	4062	4369	4563	4739
285	2963	3077	3214	3457	3747	4062	4369	4563	4739
286	2963	3077	3214	3457	3747	4062	4369	4563	4739
287	3075	3198	3346	3609	3926	4270	4606	4819	5011
288	3075	3198	3346	3609	3926	4270	4606	4819	5011
289	3075	3198	3346	3609	3926	4270	4606	4819	5011
290	3075	3198	3346	3609	3926	4270	4606	4819	5011
291	3075	3198	3346	3609	3926	4270	4606	4819	5011
292	3075	3198	3346	3609	3926	4270	4606	4819	5011
293	3075	3198	3346	3609	3926	4270	4606	4819	5011
294	3164	3297	3456	3740	4083	4456	4823	5056	5267

Mean and variance equation for male and female fetuses:

$E(Z_i) = -2.796656251349332 + [1.965246568058469\ GA_i^{0.5}] + [-0.000021935076611\ GA_i^{3}]$

$Var(Z_i) = 0.0191773432342972 + [0.0017826895572236\ GA_i] + [-0.01077702329383\ GA_i^{0.5}] + [2.38595060716e\text{-}07\ GA_i^{3}] + [-9.91625483550e\text{-}08\ GA_i^{0.5} GA_i^{3}] + [4.12225895831e\text{-}12\ GA_i^{6}]$

Supplemental table 2a. Medians and standard deviations (SD) of estimated fetal weight in grams by gestational age (GA) for male fetuses.

GA (weeks*)	-3 SD	-2 SD	-1 SD	Median	+1 SD	+2 SD	+3 SD
12	45	47	50	53	56	59	63
13	58	62	65	69	73	78	82
14	75	80	84	89	95	101	107
15	95	101	108	114	122	129	137
16	120	128	136	145	154	164	175
17	150	160	170	181	193	206	220
18	185	197	211	225	241	257	275
19	226	242	259	277	297	317	340
20	274	294	315	338	362	389	417
21	330	354	381	409	439	472	507
22	393	423	456	491	528	569	612
23	466	503	542	584	630	680	733
24	548	592	639	691	746	806	871
25	640	692	749	811	877	949	1027
26	741	804	871	944	1023	1109	1202
27	853	926	1006	1092	1185	1287	1397
28	975	1060	1153	1253	1363	1482	1611
29	1107	1205	1312	1429	1556	1694	1845
30	1247	1360	1483	1617	1764	1924	2098
31	1395	1524	1664	1818	1986	2169	2370
32	1549	1695	1855	2029	2220	2429	2658
33	1709	1873	2052	2249	2465	2702	2961
34	1870	2053	2255	2476	2718	2985	3277
35	2031	2235	2459	2706	2977	3275	3604
36	2189	2414	2662	2936	3238	3571	3938
37	2341	2588	2862	3164	3498	3867	4275
38	2483	2753	3053	3385	3753	4161	4613
39	2612	2905	3232	3595	3999	4448	4947
40	2724	3041	3395	3791	4232	4724	5274
41	2817	3158	3540	3968	4448	4986	5590
42	2886	3251	3661	4123	4643	5230	5890

*GA expressed as completed gestational weeks, e.g. 12 weeks corresponds to 12+0 weeks or 84 gestational days.

Mean and variance equation for male fetuses:

$E(Z_i) = -2.851841338008912 + [1.97964001185311\ GA_i^{0.5}] + [-0.0000223157896809\ GA_i^{3}]$

$Var(Z_i) = 0.0218178959513984 + [0.001776242639136\ GA_i] + [-0.0114833388304094\ GA_i^{0.5}] + [3.02157925386e\text{-}07\ GA_i^{3}] + [-1.04898329516e\text{-}07\ GA_i^{0.5} GA_i^{3}] + [3.66322749297e\text{-}12\ GA_i^{6}]$

Supplemental table 2b. Medians and percentiles of estimated fetal weight in grams by gestational age (GA) for male fetuses.

GA (weeks*)	2.5th	5th	10th	25th	Median	75th	90th	95th	97.5th
12	47	48	49	51	53	55	57	58	59
13	62	63	64	67	69	72	75	76	78
14	80	81	83	86	89	93	97	99	100
15	102	104	106	110	114	119	124	126	129
16	128	131	134	139	145	151	157	160	164
17	160	163	167	174	181	189	197	202	206
18	198	202	207	215	225	235	245	251	256
19	242	248	254	265	277	290	302	310	317
20	295	301	309	322	338	354	370	379	388
21	355	363	373	390	409	429	448	460	471
22	425	435	446	467	491	516	539	554	567
23	504	516	531	555	584	615	644	662	678
24	594	608	626	656	691	728	763	784	804
25	694	712	733	769	811	855	897	923	946
26	806	827	851	894	944	997	1047	1078	1105
27	929	954	983	1033	1092	1154	1213	1250	1282
28	1064	1092	1126	1185	1253	1326	1395	1438	1477
29	1209	1242	1281	1349	1429	1513	1594	1644	1688
30	1365	1402	1447	1526	1617	1715	1808	1865	1917
31	1529	1572	1623	1713	1818	1930	2036	2102	2162
32	1701	1750	1808	1910	2029	2156	2277	2353	2420
33	1879	1934	2000	2115	2249	2393	2530	2615	2692
34	2061	2123	2196	2324	2476	2637	2791	2887	2974
35	2243	2312	2394	2537	2706	2886	3058	3166	3263
36	2424	2500	2590	2749	2936	3136	3328	3449	3557
37	2599	2682	2782	2957	3164	3385	3598	3732	3851
38	2765	2856	2965	3157	3385	3628	3863	4011	4143
39	2918	3017	3136	3346	3595	3862	4120	4283	4429
40	3055	3163	3292	3520	3791	4083	4365	4543	4704
41	3172	3288	3427	3674	3968	4286	4594	4788	4964
42	3266	3391	3540	3806	4123	4467	4802	5013	5205

*GA expressed as completed gestational weeks, e.g. 12 weeks corresponds to 12+0 weeks or 84 gestational days.

Mean and variance equation for male fetuses:

$E(Z_i) = -2.851841338008912 + [1.97964001185311\ GA_i^{0.5}] + [-0.0000223157896809\ GA_i^{3}]$

$Var(Z_i) = 0.0218178959513984 + [0.001776242639136\ GA_i] + [-0.0114833388304094\ GA_i^{0.5}] + [3.02157925386e\text{-}07\ GA_i^{3}] + [-1.04898329516e\text{-}07\ GA_i^{0.5} GA_i^{3}] + [3.66322749297e\text{-}12\ GA_i^{6}]$

Supplemental table 3a. Medians and standard deviations (SD) of estimated fetal weight in grams by gestational age (GA) for female fetuses.

GA (weeks*)	-3 SD	-2 SD	-1 SD	Median	+1 SD	+2 SD	+3 SD
12	45	48	51	53	56	60	63
13	59	62	66	70	74	78	83
14	75	80	85	90	95	101	107
15	96	102	108	115	122	129	137
16	120	127	136	144	154	164	174
17	148	158	169	180	193	205	219
18	182	195	209	223	239	255	273
19	222	238	256	274	294	315	338
20	269	289	310	333	358	385	413
21	322	347	374	402	433	466	502
22	383	414	446	482	520	561	606
23	453	490	530	573	619	670	724
24	531	575	624	676	732	794	860
25	618	671	729	792	860	933	1014
26	715	778	846	921	1002	1090	1186
27	820	894	975	1063	1159	1264	1378
28	935	1022	1116	1220	1332	1456	1591
29	1058	1159	1269	1389	1521	1665	1823
30	1190	1305	1432	1572	1724	1892	2076
31	1328	1460	1606	1766	1942	2136	2349
32	1471	1621	1788	1971	2173	2395	2641
33	1617	1788	1976	2184	2415	2669	2950
34	1765	1957	2169	2405	2666	2955	3276
35	1912	2126	2365	2629	2924	3251	3615
36	2055	2293	2559	2855	3186	3555	3966
37	2192	2455	2749	3079	3449	3863	4327
38	2318	2607	2932	3298	3709	4172	4692
39	2431	2747	3104	3508	3964	4479	5060
40	2529	2872	3262	3705	4207	4778	5427
41	2608	2979	3402	3885	4437	5068	5788
42	2666	3064	3520	4045	4649	5342	6139

*GA expressed as completed gestational weeks, e.g. 12 weeks corresponds to 12+0 weeks or 84 gestational days.

Mean and variance equation for female fetuses:

$E(Z_i) = -2.732761682497818 + [1.948352785876517\ GA_i^{0.5}] + [-0.000021442768948\ GA_i^{3}]$

$Var(Z_i) = 0.0144842305561044 + [0.0014414220978001\ GA_i] + [-0.0082867824622078\ GA_i^{0.5}] + [1.76416758202e\text{-}07\ GA_i^{3}] + [-7.71173334902e\ \text{-}08\ GA_i^{0.5} GA_i^{3}] + [4.00059912927e\text{-}12\ GA_i^{6}]$

Supplemental table 3b. Medians and percentiles of estimated fetal weight in grams by gestational age (GA) for female fetuses.

GA (weeks*)	2.5th	5th	10th	25th	Median	75th	90th	95th	97.5th
12	48	49	50	52	53	55	57	58	59
13	63	64	65	67	70	72	75	76	78
14	80	82	83	86	90	93	97	99	101
15	102	104	106	110	115	119	124	126	129
16	128	130	133	138	144	151	157	160	163
17	159	162	166	173	180	188	196	201	205
18	196	200	205	213	223	234	243	249	255
19	239	244	251	261	274	287	299	307	314
20	290	296	304	318	333	350	365	375	384
21	348	356	366	383	402	423	442	454	465
22	415	425	437	458	482	507	531	546	559
23	491	503	518	543	573	604	633	651	668
24	577	592	610	640	676	713	749	771	791
25	673	691	712	749	792	837	880	906	930
26	780	801	826	870	921	975	1026	1058	1086
27	898	922	952	1003	1063	1127	1188	1226	1260
28	1025	1054	1089	1149	1220	1295	1366	1411	1451
29	1163	1197	1237	1307	1389	1477	1560	1613	1659
30	1310	1349	1395	1476	1572	1673	1770	1831	1885
31	1466	1510	1563	1656	1766	1883	1995	2065	2128
32	1628	1679	1739	1845	1971	2105	2233	2314	2386
33	1795	1853	1921	2042	2184	2337	2484	2576	2658
34	1965	2030	2107	2243	2405	2578	2744	2849	2943
35	2135	2208	2295	2448	2629	2824	3013	3131	3237
36	2303	2384	2481	2652	2855	3074	3286	3419	3539
37	2466	2555	2663	2853	3079	3324	3561	3711	3845
38	2619	2718	2837	3047	3298	3570	3834	4002	4152
39	2761	2869	2999	3231	3508	3809	4102	4288	4457
40	2887	3005	3147	3400	3705	4037	4361	4567	4754
41	2994	3122	3277	3552	3885	4249	4607	4834	5041
42	3081	3219	3385	3684	4045	4443	4835	5085	5312

*GA expressed as completed gestational weeks, e.g. 12 weeks corresponds to 12+0 weeks or 84 gestational days.

Mean and variance equation for female fetuses:

$E(Z_i) = -2.732761682497818 + [1.948352785876517\ GA_i^{0.5}] + [-0.000021442768948\ GA_i^3]$

$Var(Z_i) = 0.0144842305561044 + [0.0014414220978001\ GA_i] + [-0.0082867824622078\ GA_i^{0.5}] + [1.76416758202e\text{-}07\ GA_i^3] + [-7.71173334902e\ \text{-}08\ GA_i^{0.5} GA_i^3] + [4.00059912927e\text{-}12\ GA_i^6]$

Supplemental table 4a. Medians and standard deviations (SD) of estimated fetal weight in grams by gestational age (GA) for male and female fetuses. The table only includes subjects with BMI 18.5 to 29.9 kg/m^2.

GA (weeks*)	-3 SD	-2 SD	-1 SD	Median	+1 SD	+2 SD	+3 SD
12	45	48	50	53	56	59	62
13	59	62	66	69	73	78	82
14	76	80	85	90	95	101	106
15	96	102	108	114	121	129	137
16	120	128	136	145	154	163	174
17	149	159	170	181	193	205	219
18	184	196	210	224	239	256	273
19	225	240	257	275	295	316	338
20	272	292	313	335	360	386	414
21	326	351	377	405	436	468	504
22	389	419	451	486	524	564	607
23	460	496	536	578	624	674	727
24	540	584	632	683	738	798	863
25	630	682	739	801	867	939	1018
26	729	791	858	932	1011	1097	1191
27	838	911	990	1077	1170	1273	1384
28	956	1041	1134	1235	1345	1465	1596
29	1083	1182	1290	1408	1536	1676	1829
30	1219	1333	1457	1593	1741	1903	2081
31	1362	1492	1634	1790	1960	2147	2352
32	1510	1658	1820	1997	2192	2406	2641
33	1663	1829	2012	2213	2435	2678	2946
34	1817	2003	2209	2436	2686	2962	3266
35	1970	2178	2408	2662	2943	3254	3598
36	2119	2350	2606	2889	3204	3553	3939
37	2262	2516	2799	3114	3464	3854	4287
38	2394	2673	2985	3332	3721	4154	4638
39	2514	2818	3159	3541	3970	4450	4988
40	2616	2946	3318	3736	4207	4737	5334
41	2700	3055	3458	3913	4428	5012	5672
42	2761	3142	3576	4069	4630	5269	5996

*GA expressed as completed gestational weeks, e.g. 12 weeks corresponds to 12+0 weeks or 84 gestational days.

Mean and variance equation for male and female fetuses:

$E(Z_i)$ = -2.794531087012328 + [1.964543729452792 $GA_i^{0.5}$] + [-0.0000219469581344 GA_i^3]

$Var(Z_i)$ = 0.0150804792879315 + [0.001453830200201 GA_i] + [-0.0085235286061312 $GA_i^{0.5}$] + [1.99193180525e-07 GA_i^3] + [-8.23912408306e -08 $GA_i^{0.5}GA_i^3$] + [3.75260972756e-12 GA_i^6]

Supplemental table 4b. Medians and percentiles of estimated fetal weight in grams by gestational age (GA) for male and female fetuses. The table only includes subjects with BMI 18.5 to 29.9 kg/m^2.

GA (weeks*)	2.5th	5th	10th	25th	Median	75th	90th	95th	97.5th
12	48	49	50	51	53	55	57	58	59
13	62	63	65	67	69	72	75	76	77
14	80	82	83	86	90	93	96	98	100
15	102	104	106	110	114	119	123	126	129
16	128	131	134	139	145	151	156	160	163
17	160	163	167	173	181	189	196	201	205
18	197	201	206	214	224	234	244	250	255
19	241	246	252	263	275	288	300	308	315
20	292	299	307	320	335	352	367	377	385
21	352	360	370	386	405	426	445	457	467
22	420	430	442	462	486	511	535	549	562
23	498	510	524	549	578	609	638	656	671
24	586	601	618	648	683	720	755	777	796
25	684	702	722	759	801	845	887	913	936
26	794	814	839	882	932	984	1035	1066	1094
27	914	938	967	1018	1077	1139	1198	1235	1268
28	1045	1073	1107	1166	1235	1309	1378	1422	1460
29	1186	1219	1259	1327	1408	1493	1574	1625	1670
30	1337	1376	1421	1500	1593	1691	1785	1844	1897
31	1497	1541	1592	1683	1790	1903	2011	2079	2139
32	1664	1713	1772	1876	1997	2126	2250	2328	2397
33	1836	1892	1959	2075	2213	2360	2501	2589	2668
34	2011	2074	2149	2280	2436	2602	2761	2861	2950
35	2187	2257	2341	2488	2662	2849	3028	3140	3241
36	2360	2438	2531	2695	2889	3098	3299	3425	3538
37	2527	2613	2716	2898	3114	3346	3570	3711	3837
38	2685	2780	2893	3094	3332	3589	3838	3995	4136
39	2831	2934	3059	3279	3541	3824	4099	4273	4430
40	2960	3073	3208	3449	3736	4047	4350	4542	4715
41	3071	3193	3339	3600	3913	4253	4586	4796	4987
42	3159	3290	3448	3730	4069	4439	4802	5033	5242

*GA expressed as completed gestational weeks, e.g. 12 weeks corresponds to 12+0 weeks or 84 gestational days.

Mean and variance equation for male and female fetuses:

$E(Z_i)$ = -2.794531087012328 + [1.964543729452792 $GA_i^{0.5}$] + [-0.0000219469581344 GA_i^3]

$Var(Z_i)$ = 0.0150804792879315 + [0.001453830200201 GA_i] + [-0.0085235286061312 $GA_i^{0.5}$] + [1.99193180525e-07 GA_i^3] + [-8.23912408306e -08 $GA_i^{0.5}GA_i^3$] + [3.75260972756e-12 GA_i^6]
